# Discriminative Analysis of Different Grades of Gaharu (*Aquilaria malaccensis* Lamk.) via ^1^H-NMR-Based Metabolomics Using PLS-DA and Random Forests Classification Models

**DOI:** 10.3390/molecules22101612

**Published:** 2017-09-25

**Authors:** Siti Nazirah Ismail, M. Maulidiani, Muhammad Tayyab Akhtar, Faridah Abas, Intan Safinar Ismail, Alfi Khatib, Nor Azah Mohamad Ali, Khozirah Shaari

**Affiliations:** 1 Laboratory of Natural Products, Institute of Bioscience, Universiti Putra Malaysia, 43400 Serdang, Malaysia; sitinazirahismail12@yahoo.com (S.N.I.); dieni_maulydia@yahoo.com (M.M.); tayyabakhtar@hotmail.com (M.T.A.); faridah_abas@upm.edu.my (F.A.); safinar@upm.edu.my (I.S.I.); 2 Department of Food Science, Faculty of Food Science and Technology, Universiti Putra Malaysia, 43400 Serdang, Malaysia; 3 Department of Chemistry, Faculty of Science, Universiti Putra Malaysia, 43400 Serdang, Malaysia; 4 Department of Pharmaceutical Chemistry, Kuliyyah of Pharmacy, International Islamic University Malaysia (Kuantan Campus), Bandar Indera Mahkota, 25200 Kuantan, Malaysia; alfikhatib@iium.edu.my; 5 Forest Research Institute Malaysia, 52109 Kepong, Malaysia; norazah@frim.gov.my

**Keywords:** gaharu, *Aquilaria malaccensis*, quality, NMR-based metabolomics, PLS-DA, Random Forests classifier

## Abstract

Gaharu (agarwood, *Aquilaria malaccensis* Lamk.) is a valuable tropical rainforest product traded internationally for its distinctive fragrance. It is not only popular as incense and in perfumery, but also favored in traditional medicine due to its sedative, carminative, cardioprotective and analgesic effects. The current study addresses the chemical differences and similarities between gaharu samples of different grades, obtained commercially, using ^1^H-NMR-based metabolomics. Two classification models: partial least squares-discriminant analysis (PLS-DA) and Random Forests were developed to classify the gaharu samples on the basis of their chemical constituents. The gaharu samples could be reclassified into a ‘high grade’ group (samples A, B and D), characterized by high contents of kusunol, jinkohol, and 10-epi-γ-eudesmol; an ‘intermediate grade’ group (samples C, F and G), dominated by fatty acid and vanillic acid; and a ‘low grade’ group (sample E and H), which had higher contents of aquilarone derivatives and phenylethyl chromones. The results showed that ^1^H- NMR-based metabolomics can be a potential method to grade the quality of gaharu samples on the basis of their chemical constituents.

## 1. Introduction

*Aquilaria* Lam. (family Thymelaeaceae), is a genus with 22 accepted species [1], distributed mainly in the tropical forest of South-East Asian countries, including Malaysia [2,3]. Species of this genus are the principal source of gaharu (also known as agarwood, aloeswood or eaglewood) which is one of the most valuable forest products, traded internationally for centuries. Gaharu refers to the fragrant, dark and dense, resinous heartwood, pathologically formed in response to injury and microbial infection [4,5,6]. This forest product is not only popular as incense and in perfumery, but it is also favored in traditional medicine, with sedative, carminative, cardioprotective and analgesic effects reported to be among the ethnopharmacological properties associated with it [7,8]. *Aquilaria* species that have been reported to produce valuable gaharu include *A. malaccensis* Lamk., *A. crassna* Pierre ex Lecomte, *A. beccariana* Tiegh., *A. hirta* Ridl., *A. rostrata* Ridl., *A. sinensis* (Lour.) Spreng., *A. microcarpa* Baill., *A. filaria* (Oken) Merr. and *A. khasiana* Hallier f. [9]. By far, *A. malaccensis* or kekaras (in Malay), is the most common gaharu-producing species in Malaysia. The species has been well recognized for its commercial value, representing an important export commodity to the Middle East countries, Japan and China [4,10,11].

Gaharu is traded in the form of wood chips, sawn wood, and resin or distilled oil. In trade, the selling price varies greatly, depending on the ‘quality’ [12,13]. The quality of gaharu is usually graded based on its physical properties such as wood color, weight or density, and aroma upon burning, all of which, indirectly reflect the resin content of the gaharu samples. High quality gaharu would have higher content of resin, would be of higher density, and would have darker color and stronger aroma [3,14,15]. In the Malaysian gaharu market, the ABC Agarwood Grading System [11,16], which is highly based on physical characteristics, still remains as the most common method of grading gaharu. The system is widely used by traders, despite various efforts to develop a more viable and scientific method of grading [17,18,19,20]. However, relying on physical properties as a means of grading has many drawbacks since it is highly dependent on individual human perceptions, possibly resulting in bias and poor reproducibility. The subjective nature of the existing grading systems and lack of standard method of grading have contributed to a high incidence of adulteration and substitution in the gaharu trade [4,10]. Thus, an analysis based on the inherent chemical characteristics of the different types or class of gaharu would provide a more accurate method of their quality assessment. High grade gaharu samples are reported to have high contents of sesquiterpenes [12,14,21,22,23]. Other studies also showed that high grade gaharu contained high levels of 10-epi-γ-eudesmol, aromadendrane, β-agarofuran, α-agarofuran, γ-eudesmol, epoxybulnesene and α-guaiene [24,25,26,27]. Meanwhile, Hung et al. [28] found that α-copaene, *trans*-caryophyllene, and δ-guaiene were present in the most expensive agarwood powders/extracts. Although these studies have provided useful insights into the chemical profile of gaharu, more information is needed in order to make meaningful correlations between chemical constituents and the quality of gaharu.

Metabolomics is an approach used to study the global profile of chemical constituents of an organism. It combines the use of analytical measurements (e.g., FTIR, ^1^H-NMR, GC-MS, LC-MS) and multivariate data analysis to classify and identify metabolites in biological samples [29,30,31,32]. Previously, metabolomics has been successfully used to classify different grades of agarwood powder [28,33,34] and oils [34,35,36]. The analytical tools used in these studies were mainly GC-MS [33,34,35], GC-MS coupled with solid-phase microextraction (SPME) [22,24,25,27,28] and two-dimensional GC coupled to accurate mass time-of-flight mass spectrometry (TOFMS) [36]. Although GC-MS can identify more metabolites in comparison to ^1^H-NMR spectroscopy, the identification of sesquiterpenoids (major component in agarwood essential oil) in gaharu samples using GC-MS alone may lead to errors [14]. It is important that these results be supported and substantiated with other complementary data using other spectroscopic techniques. Although less sensitive, ^1^H-NMR spectroscopy is fast and highly reproducible as well as requiring simple and non-destructive sample preparation. The technique can measure a wide range of metabolites [37,38] and is a method of choice for many metabolomics studies [30]. To date, the application of ^1^H-NMR-based metabolomics to differentiate various grades of gaharu based on their metabolite profiles has not been attempted. It is the objective of the present study to obtain a better insight into the chemical constituents of the various grades of gaharu. The study aims to ascertain if the different grades can be differentiated based on their metabolite profiles and whether the chemical information can be used as a means to grade the quality of gaharu.

## 2. Results and Discussion

### 2.1. Identification of Gaharu Metabolites

**Figure 1:**
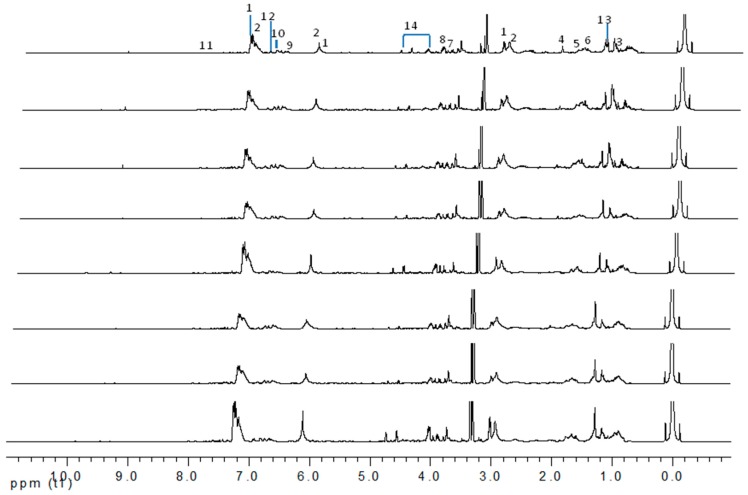
^1^H-NMR spectra of eight groups (A-H) of *A*. *malaccensis* gaharu samples of different grades. Metabolites labeled with numbers were tentatively identified. The spectra are arranged according to the selling price of the gaharu samples, from grades A (the most expensive) through B, C, D, E, F, G to H (the cheapest), respectively.

Figure 1 shows representative ^1^H-NMR spectra of the methanol extracts of gaharu samples obtained from Malaysia. The samples were of varying qualities and were grouped according to their selling price (Supplementary Table S1). Visual inspection of the spectra indicated that the samples have very similar chemical profiles, with the expected differences in the concentration of the individual constituents. The main chemical shifts identified from the ^1^H-NMR spectra were in the regions of aromatics (δ 6.00–7.50), sugars and glycosides (δ 2.50–5.00), and fatty acids/aliphatics (δ 0.50–2.00). The complete ^1^H-NMR assignments for the identified metabolites are tabulated in Table 1.

The representative signals of two phenylethyl chromones i.e., 6-hydroxy-2-(2-phenylethyl)-chromone (6HC, **1**) and 6-hydroxy-2-[2-(4-hydroxyphenyl)ethyl]chromone (6DHC, **2**) were observed in the ^1^H-NMR spectra of the gaharu extracts. Compound **1** was identified based on the ^1^H-NMR signals for an ABX coupling system at δ 7.14 (1H, d, *J* = 8.5 Hz, H-8), 6.83 (1H, dd, *J* = 8.5, 2.5 Hz, H-7), and 8.09 (1H, d, *J* = 2.5 Hz, H-5) and a singlet proton at δ 6.10 (1H, s, H-3), characteristic of the chromone benzopyran moiety. In addition, signals for the two sets of methylene protons for the phenylethyl moiety were also observed at δ 3.02 (2H, m) and 2.92 (2H, m). The identification of compound **1** was further supported by 2D-NMR experiments (*J*-resolved, COSY, and HMBC spectra), LC-MS/MS analysis (data shown in Supplementary Table S2) and comparison with literature values [39]. Compound **2** was similarly assigned based on signals observed at 6.62 (1H, d, *J* = 8.0 Hz, H-8), 6.73 (1H, dd, *J* = 8.0, 2.5 Hz, H-7) and δ 7.98 (1H, d, *J* = 2.5 Hz, H-5) for the ABX coupled protons, singlet proton at δ 6.11 (1H, s, H-3) and the two sets of methylene protons at δ 2.95 (2H, m) and 2.89 (2H, m). The presence of an A_2_B_2_ coupled system at δ 7.21 (2H, d, *J* = 8.0 Hz) and 7.17 (2H, d, *J* = 8.0 Hz) supported the *para*-hydroxyphenyl ring of the phenylethylchromone structure.

Besides the signals for the phenylethylchromones, signals attributable to 5,6,7,8-tetrahydro-chromone (**14**) were also observed at δ 4.72 (d), 4.55 (d), 4.29 (m), 3.99 (dd) and two sets of methylene protons at δ 2.70–2.80 (m). Comparison with literature values [40] and a molecular ion peak observed at *m/z* 363 [M − H]^−^ in the LC-MS spectrum (Supplementary Table S2), supported the tentative identification of the compound as an aquilarone derivative. Minor constituents from the phenolic class of compounds were also identified in the gaharu samples, tentatively assigned as vanillic acid (**8**), cinnamic acid (**9**), *o*-cresol (**10**), xanthosine (**11**) and catechol (**12**) (Table 1).

Sesquiterpenes have been reported to be a major class of compounds present in the resin of *A. malaccensis* [13]. Yoneda et al. [41] also reported the presence of jinkohol, kusunol, α-agarofuran, 10-epi-γ-eudesmol, and agarospirol in the agarwood oil obtained from *A*. *malacensis*. After detailed analysis and a comparison with literature data and online databases, the sesquiterpenoids jinkohol (**3**) and kusunol (**4**) [42], agarofuran (**5**) and epieudesmol (**6**) [43], and isoeugenol (**7**) (HMBD http://www.hmdb.ca/) were also identified in the gaharu extracts in the present study. Further examination of the ^1^H-NMR spectra also showed the presence of very small amounts of aldehydic compounds (δ 9.32 ppm) as can be seen in Figure 1. However, due to technical limitations of the present study, the structures of these aldehydes could not be identified.

### 2.2. Discriminative Analysis of Gaharu Samples

The processed ^1^H-NMR data was initially subjected to principal component analysis (PCA) in order to see the differences between the eight groups of gaharu. However, PCA did not show any clear clustering or differences among the gaharu samples. This could be due to the high variability of the different gaharu samples. Partial least squares-discriminant analysis (PLS-DA) was then used to model the relationships between the eight groups of gaharu samples. A permutation test was applied to evaluate the reliability of the model (Supplementary Figure S1). Overall, the PLS-DA model was found to be a reliable and good model for the classification. The model did not show over-fitting, based on the *Y*-axis intercept values of R^2^ = 0.07 and Q^2^ = −0.14, and the fact that the R^2^ line was far from being horizontal.

**Figure 2:**
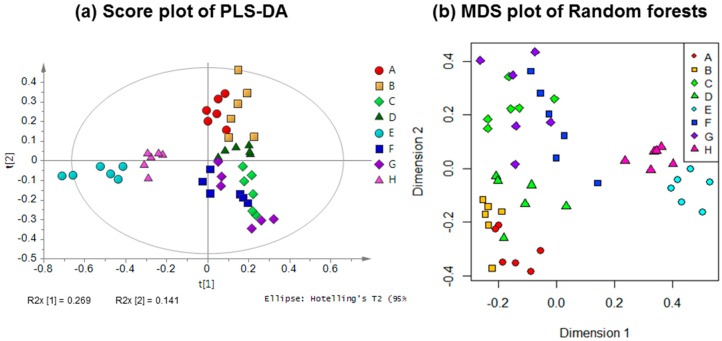
(**a**) PLS-DA score plot and (**b**) Random Forests multidimensional scaling (MDS) plot for *A. malaccensis* gaharu samples of different grades.

**Figure 3:**
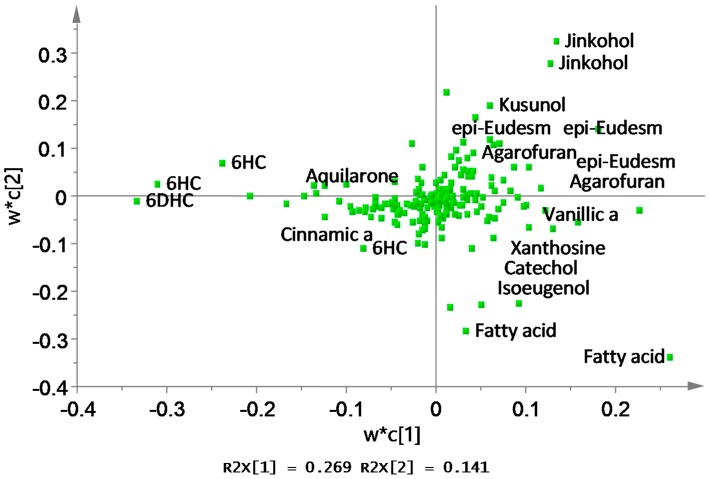
Loading plots corresponding to the PLS-DA score plot.

The PLS-DA score plot showed that the eight groups of gaharu samples were differentiated into three distinct clusters (Figure 2a). The samples H and E were well separated from the rest of the samples (A, B, C, D, F, G), and were clustered together on the negative side of PLS component 1. Meanwhile, samples A, B, C, D, F, G could be further differentiated on the basis of PLS component 2 scores, where samples A, B and D were clustered on the positive side, while samples C, F and G were clustered on the negative side. The corresponding loading plot (Figure 3) showed the discriminant metabolites responsible for the separation of the three clusters in the score plot. From the loadings, it could be deduced that samples H and E contained higher levels of 6-hydroxy-2-(2-phenylethyl)-chromone (6HC, **1**), 6-hydroxy-2-[2-(4-hydroxyphenyl)ethyl]chromone (6DHC, **2**), cinnamic acid (**9**) and aquilarone derivatives (**14**). The samples A, B and D were marked by higher levels of jinkohol (**3**), kusunol (**4**), agarofuran (**5**), and 10-epi-γ-eudesmol (**6**), whereas C, F and G were characterized by higher levels of isoeugenol (**7**), vanillic acid (**8**), xanthosine (**11**), catechol (**12**) and fatty acids (**13**). A blind test was carried out to evaluate the performance of the PLS-DA model (Supplementary Figure S2). A new batch of gaharu samples (test samples) belonging to low, medium and high grades were analyzed by ^1^H-NMR and subjected to PLS-DA together with the training set (previous NMR data of the different grades). In the PLS-DA score plot, the new gaharu samples were clustered well within the corresponding grades.

To further validate the results obtained from the PLS-DA, the Random Forests classifier was applied to the same ^1^H-NMR data. In contrast to PLS-DA, the application of Random Forests as a classification model [44] is relatively rare in metabolomics data analysis. Although it is available in freeware softwares such as the Random Forest package in the R software [45] and MetaboAnalyst [46], its applicability in metabolomics studies still needs to be explored. Figure 2b shows the Random Forests multi-dimensional scaling (MDS) plot of proximity matrix. The MDS plot showed the same clustering as was found in the score plot of PLS-DA. The accuracy of the models was evaluated using confusion matrices as shown in Table 2.

The percentage of overall agreement (given by 100∑Xii)/N) and Kappa coefficient or κ (given by [(∑Xii) − (∑Xi + X + i)/N]/[N − (∑Xi + X + i)/N]) values were calculated to be 72.9% and 0.69, respectively.

### 2.3. Identification of Discriminating Metabolites

**Figure 4:**
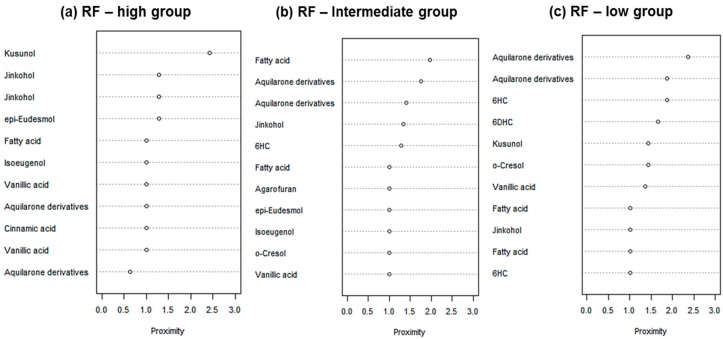
Variable importance (VIP) values for (**a**) high; (**b**) intermediate and (**c**) low grade clusters based on Random Forests.

The discriminating metabolites were identified from the chemical shifts in the PLS-DA loading plot (Figure 3), and from the VIP (variable importance) values in the Random Forests for each cluster of gaharu samples (Figure 4). In the latter, metabolites having high value of VIP are deemed to have high contribution to the clustering.

For the PLS-DA model, the high grade cluster (groups A, B and D) was characterized by higher levels of jinkohol (**3**), kusunol (**4**), and 10-epi-γ-eudesmol (**6**), whereas the intermediate grade cluster (groups C, F and G) contained higher levels of isoeugenol (**7**), vanillic acid (**8**), xanthosine (**11**), catechol (**12**) and fatty acid (**13**). The low grade cluster (groups H and E) was distinguished from the other groups by having higher levels of aquilarone derivatives (**14**) and phenylethylchromones **1** and **2**. The Random Forests analysis basically resulted in the identification of the same discriminant metabolites in the established clusters as in the PLS-DA model.

Identification of the discriminant metabolites from the PLS-DA and Random Forests models were confirmed by analysis of the variable importance (VIP) values for all clusters as shown in the Supplementary Figure S3a,b, respectively. Furthermore, the relative quantification of the discriminant metabolites in the three clusters (high, intermediate and low grade clusters) was carried out using Tukey posthoc analysis, based on the average peak area of the corresponding ^1^H-NMR signals (Supplementary Figure S4).

The sesquiterpenoids jinkohol (**3**), kusunol (**4**), α-agarofuran (**5**) and 10-epi-γ-eudesmol (**6**) are well known volatile constituents that are associated with the fragrance of gaharu [14,26]. In the present study, the high grade gaharu samples were indeed characterized by high levels of jinkohol (**3**), kusunol (**4**) and 10-epi-γ-eudesmol (**6**), and thus, were in agreement with previous findings [14,15,21]. The higher grade gaharu samples were also observably darker in color in comparison to the low grade gaharu samples which clearly reflected the higher contents of the resinous constituents. According to the literature, non-infected *A*. *malaccensis* wood is brighter in colour and almost odourless, whereas the infected wood is heavier and dark brown to black in colour [14,15]. On the other hand, chromones have been reported to be the metabolites responsible for the warm, sweet, balsamic and long-lasting odor when gaharu wood is burned or heated [14]. Therefore, the lower grade gaharu samples which were richer in these chromones are more suitable for use as incense. Jinkohol (**3**), the proposed chemical marker for high grade gaharu, also has a distinctive and extremely strong woody smell which contributed to the suitability of the high grade gaharu extract/resin as a perfume ingredient. Several studies have also reported high levels of agarofuran (**5**) in gaharu samples of high grade [24,25,26,27]. In the present study, however, both the PLS-DA and Random Forests models showed that the levels of the constituent in the different groups were not significantly different.

Interestingly, although PLS-DA and Random Forests are based on different concepts, both models yielded similar results in terms of class plots (score and MDS plots) and the chemical constituents for the new group clusters, as well as the VIP values. We noted that since Random Forests was developed based on a random subset in both variables and individual data, repeating the Random Forests analysis may not always yield exactly the same results, albeit it was similar when we rerun the analysis. However, the Random Forests result may still explain about uncertainties in the biological system.

^1^H-NMR-based metabolomics was shown to be effective in classifying *A. malaccensis* gaharu samples of varying quality, as sampled from the market place. Using this approach, it was also possible to propose a new group of classification based on their chemical constituents. Random Forests and PLS-DA were found to be reliable chemometric methods to assess the differences and similarities among the different gaharu samples. Using the two methods, the gaharu samples analysed in the present study, could be reclassified into three groups based on their chemical characteristics. From the identified gaharu constituents, eight metabolites could be proposed as differentiating chemical constituents between the high (jinkohol (**3**), kusunol (**4**), and 10-epi-γ-eudesmol (**6**)), intermediate (fatty acid (**13**) and vanillic acid (**8**)) and low (phenylethyl chromones (**1** and **2**) and aquilarone derivatives (**14**)) grades of *A. malaccensis* gaharu. However, the results were based on relative quantification of the metabolites. Further confirmatory analyses are required to determine the absolute quantification and identification of these chemical constituents.

## 3. Experimental Section

### 3.1. Samples and Chemicals

Samples of *A*. *malaccensis* gaharu in the form of wood chips were purchased from an experienced collector and trader. The samples consisted of varying quality of gaharu samples (Supplementary Table S1), graded and valued (RM/kg) by the collector according to the ABC Agarwood Grading System. The gaharu samples were inspected for authenticity and the grading was double checked and confirmed by an in-house expert. For the purpose of the present study, the samples were grouped according to their selling price and labeled A to H. Each group of gaharu samples consisted of six replicates. Lab grade methanol (redistilled prior to use), methanol-d4 (CD_3_OD, 99.8%), KH_2_PO_4_, sodium deuterium oxide (NaOD) and deuterium oxide (D_2_O) (99.8%) were purchased from Merck (Darmstadt, Germany).

### 3.2. ^1^H-NMR Sample Preparation

Each sample of gaharu wood chips was pulverized into fine powder using mortar pestle and grinder. To ensure that wood particles are of uniform size, the ground samples were sieved using sieve shaker (Retsch) to collect ≤140 μm particles. The sieved samples (1 g) were then extracted with 10 mL lab grade methanol (sample:solvent ratio of 1:6 (*w*/*v*) by sonication (Ultrasonic LC 60H, Elma, Singen, Germany), for 1 h at ambient temperature. The extracts were filtered and the collected filtrates taken to dryness under vacuum (MiVac, Genevac, Ipswich, UK), followed by lyophilization. All extracts were kept at −80 °C until further analysis.

Samples for NMR measurements were prepared by resuspending 20 mg of each sample extract in 700 µL CD_3_OD to which 0.5% tetramethylsilane (TMS) had been added as reference standard. The sample-solvent mixtures contained in 1.5 mL Eppendorf tubes were then sonicated for 15 min at room temperature to facilitate resolubilization of the extract in the NMR solvent. After centrifuging for a further 15 min at a speed of 13,000 rpm, the clear supernatant solutions of each sample (700 µL) were transferred into 5 mm NMR tubes for NMR data acquisition.

### 3.3. ^1^H-NMR Data Acquisition and Data Preprocessing

In total, 48 samples were analyzed (8 groups × 6 replicates). ^1^H-NMR spectra were recorded at 25 °C on a Unity Inova 500 MHz NMR spectrometer (Varian, Palo Alto, CA,USA) using 128 scans over a proton frequency range of 15 ppm. The PRESAT program was used to suppress undesirable signals caused by residual water. *J*-resolved and 2D NMR analysis were also performed for structural elucidation of chemical constituents present in the extract. The generated ^1^H-NMR FIDs were manually processed, phase corrected and referenced to the internal standard, TMS (δ 0.00 ppm). Baseline correction was applied to all spectra before converting to ASCII file and binned using Chenomx software after which the processed raw data was saved in an Excel spreadsheet (Microsoft, Washington, DC, USA). The raw data were binned into individual widths of δ 0.04 starting from chemical shift region δ 0.5 to δ 10.00 ppm. Water and solvent peaks in the region δ 4.70–4.96 and δ 3.28–3.33 ppm, respectively, were excluded in the multivariate analysis.

### 3.4. Metabolite Assignment

The metabolites were identified by comparing the characteristic peak signals in the ^1^H-NMR spectra of samples with published data and existing literature databases (www.hmdb.ca; Chenomx NMR Suit Ver.7.1, company Edmonton, AB, Canada). Identification of compounds was also supported by 2D NMR and LC-MS analysis. Table 1 and Supplementary Table S2 show the identified metabolites in the gaharu samples.

### 3.5. Development of PLS-DA and Random Forests Models

PLS-DA is a supervised classification technique. This technique optimizes separation between different groups of samples and develops link between two data matrices X (i.e., data, binned spectra) and Y (i.e., groups, class membership etc.) by maximizing the covariance between these X and Y matrices and finding a linear subspace of the explanatory variables [30,47]. The Y-variables are represented with a special binary ‘dummy’ [30,48]. Data were Pareto-scaled and PLS-DA was carried out using SIMCA-P software (version 12.0, Umetrics, Umea, Sweden). Random Forests is a tree-based ensemble method where two subsets are operated in independent variables at each node and in individual observation data by bootstrapping technique. Random Forests can be used for unsupervised and supervised classification as well as regression. In the current study, random Forests was used for a supervised classification, performed using the Random Forest R package [45].

### 3.6. Statistical Analysis

The relative quantification of chemical constituents was based on the mean binned peak height of the related ^1^H-NMR signals. ANOVA and Tukey’s honest multiple comparison tests were conducted to evaluate the significant difference (*p* < 0.05) between the differentiating metabolites. The statistical analysis was performed using SPSS version 16.0 (SPSS Inc., Chicago, IL, USA) software.

## 4. Conclusions

The study showed that, using ^1^H-NMR-based metabolomics, it is possible to discriminate between *A. malaccensis* gaharu samples of different quality. The results provide an insight into the chemical characteristics of gaharu, categorizing the samples into high, intermediate and low grades. Although more extensive work needs to be done, such as applying the analysis to other gaharu-producing species, the information obtained in this study is of importance and contributes towards development of a ‘chemical assay’ that could make the process of grading gaharu or agarwood samples more accurate, practical and efficient.

## Figures and Tables

**Table 1:** Identified metabolites in the ^1^H-NMR spectra of (*A. malaccensis*) gaharu samples.

Tentative Compound	Chemical Shifts
6-Hydroxy-2-(2-phenylethyl)chromone (**1**) 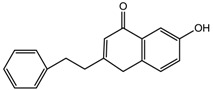	8.09 (d, *J* = 2.5 Hz, H-5); 7.26–7.19 (m, H-2′-H-6′); 7.14 (d, *J* = 8.5 Hz, H-8);6.10 (s, H-3); 3.02–2.99 (m, H-7′); 2.92–2.90 (m, H-8′)
6-Hydroxy-2-[2-(4-hydroxyphenyl)ethyl]chromone (**2**) 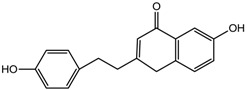	7.98 (d, *J* = 2.5 Hz, H-5); 7.21 (d, *J* = 8.0 Hz, H-2′); 7.17 (d, *J* = 8.0 Hz, H-6′); 6.73 (dd, *J* = 8.0, 2.5 Hz, H-7); 6.11 (s, H-3); 2.95 (m, H-7'); 2.87 (m, H-8')
Jinkohol (**3**) 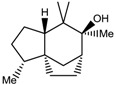	2.01 (dd, *J =* 4.9, 4.4 Hz, H-8); 1.86 (m, H-3); 1.80 (m, H-2); 1.69 (dd, *J =* 9.8 Hz, H-5); 1.56 (ddd, *J =* 10.6, 1.5 Hz, H-11); 1.38 (dd, *J =* 10.6, 4.4 Hz, H-11); 0.90 (s, 6-Me); 0.84 (d, *J* = 6.5 Hz, 2-Me)
usunol (**4**) 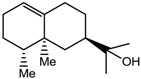	5.32 (ddd, *J =* 5.7, 2.2 Hz, H-1); 2.27 (dddd, *J* = 13.8, 12.4, 3.3 Hz, H-9); 1.62 (dddd, *J* = 12.4, 3.3 Hz, H-7); 1.41 (m, H-4)
α-Agarofuran (**5**) 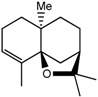	5. 59 (s, H-3); 2.22 (dd, *J =* 12.5, 4.0 Hz, H-9); 1.72 (s, H-12); 1.23 (s, H-14); 0.91 (s, H-13)
10-epi-γ-Eudesmol (**6**) 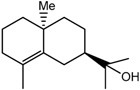	2.12 (d, *J =* 15.8 Hz, H-3); 1. 68 (s, H-12); 1.19 (s, H-13); 1.09 (s, H-11)
Isoeugenol (**7**) 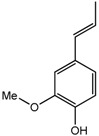	7.09 (dd, *J =* 1.9, 0.5 Hz, H-5); 3.79 (s, H-10); 1.55 (d, *J =* 7.3 Hz, H-9); 6.32 (d, *J =* 16.9 Hz, H-7); 6.29 (dq, *J =* 16.9, 6.9 Hz, H-8); 7.40 (dd, *J =* 8.6, 1.9 Hz, H-3)
Vanillic acid (**8**) 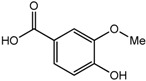	3.94 (s, H-8); 6.92 (d, *J =* 8.2 Hz, H-3); 7.43 (dd, *J =* 8.2, 1.7 Hz, H-4); 7.52 (d, *J =* 1.7 Hz, H-6)
Cinnamic acid (**9**) 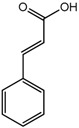	7.60 (dd, *J =* 7.9, 1.1 Hz, H-6); 7.45 (m, H-5); 7.40 (d, trans, *J =* 16.0 Hz, H-7); 6.54 (d, trans, *J =* 16.0 Hz, H-8)
*o*-Cresol (**10**) 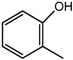	2.29 (s, H-8); 6.82 (m, H-4, H-6); 7.14 (m, H-5); 7.20 (m, H-3)
Xanthosine (**11**) 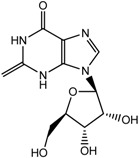	7.88 (s, H-7); 5.85 (d, *J =* 6.4 Hz, H-2); 4.69 (t, *J =* 5.7 Hz, H-3); 4.25 (q, *J =* 2.7 Hz, H-5); 3.89 (m, H-17)
Catechol (**12**) 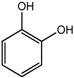	6.87 (m, H-4, H-5); 6.94 (m, H-3, H-6)
Fatty acid: (**13**)	1.28 (m)
Aquilarone derivatives (**14**) 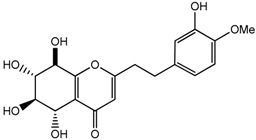	4.72 (d, *J =* 2.7 Hz, H-5); 4.55 (d, *J =* 7.3 Hz, H-8); 4.29 (m, H-6); 3.99 (dd, *J =* 6.2, 2.4 Hz, H-7); 2.70–2.80 (m, 2H)

**Table 2:** Confusion matrix for Random Forests in the classification of eight groups of *A. malaccensis* gaharu samples of different grades.

		Random Forests Class									Producer Accuracy	
		A	B	C	D	E	F	G	H	Total	Percent Correct	Omission Error (%)
Reference class	A	4	2	0	0	0	0	0	0	6	67	33
	B	2	3	0	1	0	0	0	0	6	50	50
	C	0	0	5	0	0	0	1	0	6	83	17
	D	0	2	0	3	0	1	0	0	6	50	50
	E	0	0	0	0	6	0	0	0	6	100	0
	F	0	0	0	1	0	5	0	0	6	83	17
	G	0	0	1	1	0	1	3	0	6	50	50
	H	0	0	0	0	0	0	0	6	6	100	0
	Total	6	7	6	6	6	7	4	6	48		
Users accuracy												
Percent correct		67	43	83	50	100	71	75	100		72.92	
Commission error (%)		33	57	17	50	0	29	25	0			
Agreement		4	3	5	3	6	5	3	6	35		
By chance		0.75	0.88	0.75	0.75	0.75	0.875	0.5	0.75	6.00		
	Kappa	0.69										

## Supplementary Materials

Supplementary Materials are available online.
