# Supplementary material for: Discriminative Analysis of Different Grades of Gaharu (Aquilaria malaccensis Lamk.) via 1H-NMR-Based Metabolomics Using PLS-DA and Random Forests Classification Models
Source: Molecules. 2017 Sep 25;22(10):1612. doi: 10.3390/molecules22101612 (PMC6151416; doi:10.3390/molecules22101612)
Supplement: Supplementary file 1 [file molecules-22-01612-s001.pdf]

Supplementary information

Supplementary Table 1. *A. malaccensis* gaharu samples analysed in the present study. The samples were obtained from traders in Malaysia.

Label in this study	Market label	Sample image	Price (RM/kg)
A	Kayu AAA - high grade	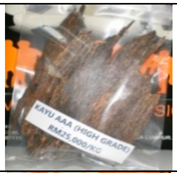	25,000
B	Kayu A - high grade	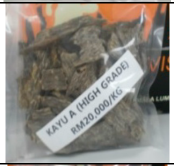	20,000
C	Teras	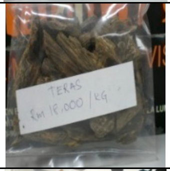	18,000
D	Kayu A - small	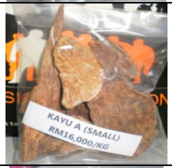	16,000
E	Kayu B - high grade	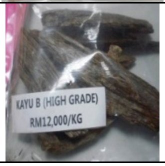	12,000

Label in this study	Market label	Sample image	Price (RM/kg)
F	Kayu A - medium	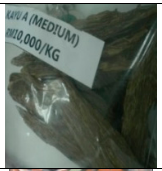	10,000
G	Kayu A - hancur	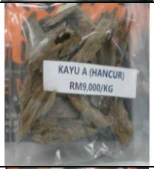	9,000
H	Teras	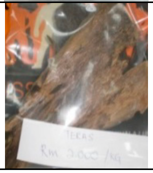	2,000

Supplementary Table. 2. LC-MS data of identified metabolites in eight gaharu samples.

No	Compound name	m/z	structure
1	6- or 7-hydroxy-2-[(2-phenyl)-ethyl]chromone ($C_{17}H_{14}O_3$	[M-H]$^-$ ion at m/z value of 265<br>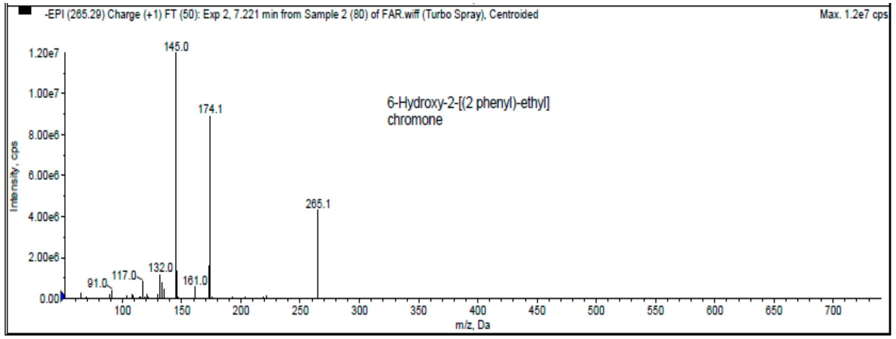	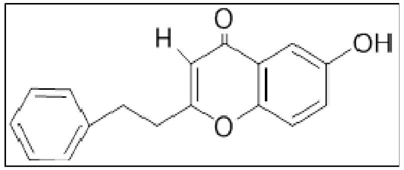
2	2 isomers of dihydroxy-2-[(2-phenyl)-ethyl]chromones	m/z values of 281<br>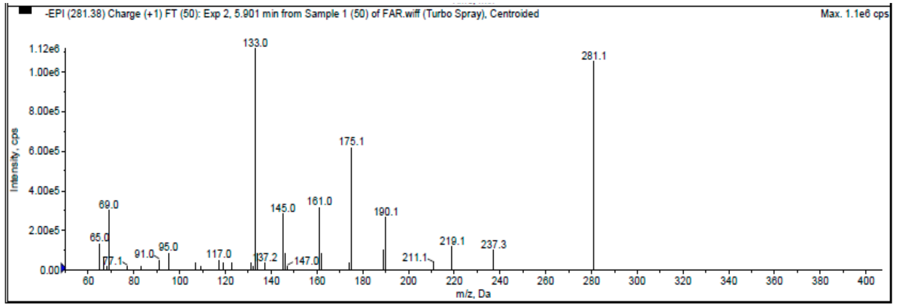<br>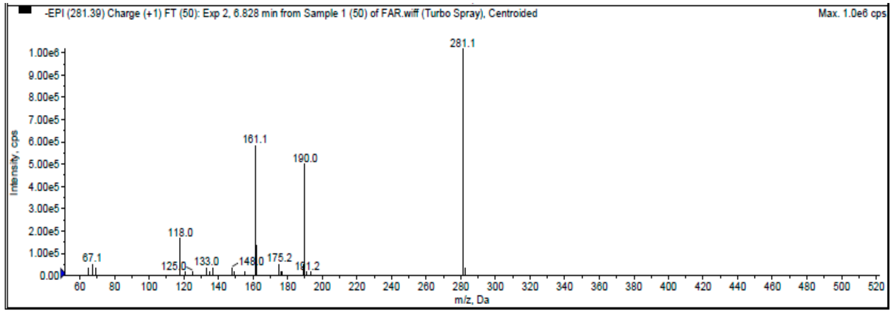	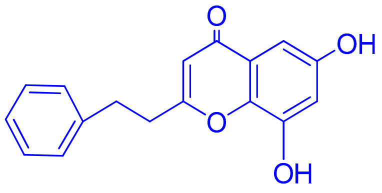<br>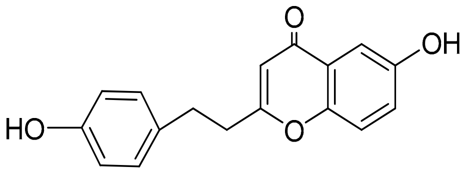

3	(*9Z,12Z*)-15,16-dihydroxyoctadeca-9,12-octadecadienoic acid	[M-H]⁻ ion of m/z 311<br>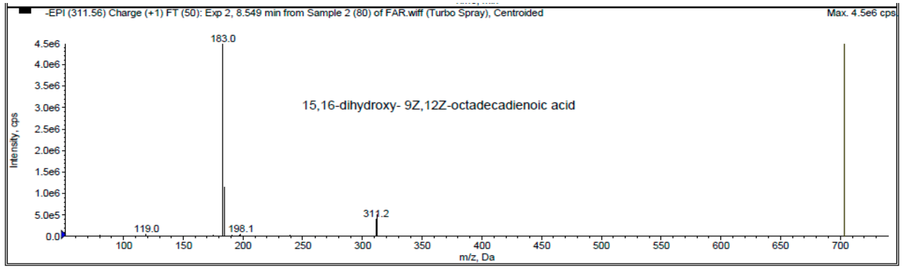 	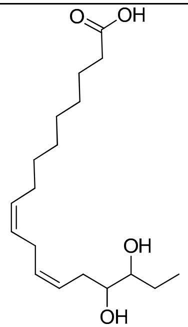
4	(*11Z,14Z*)-17,18-dihydroxyicosa-11,14-dienoic acid (m/z 340, $C_{20}H_{36}O_4$),	[M-H]⁻ ion at m/z 339<br>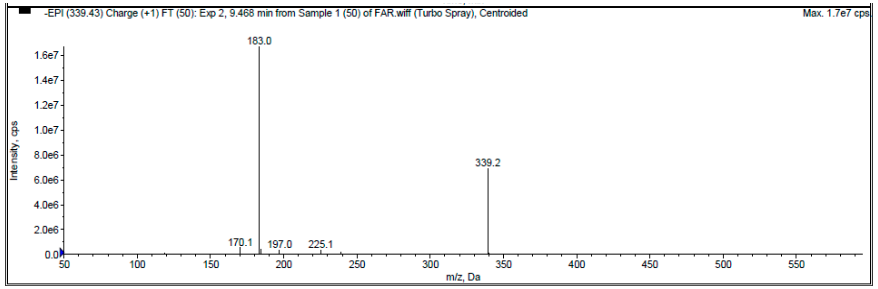 	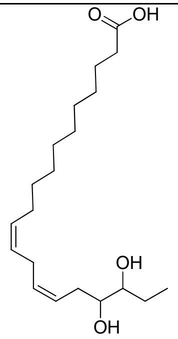
5	aquilarone F	[M-H]⁻ ions of m/z 333<br>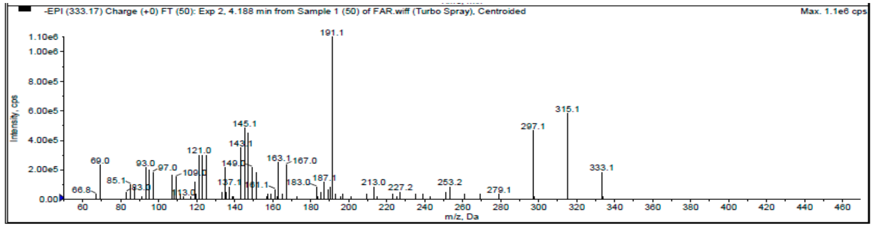 	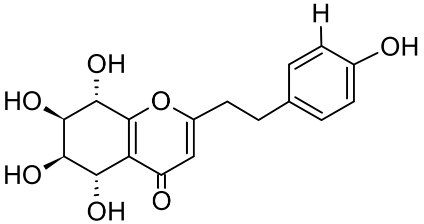

6	aquilarone D or E	[M-H]$^-$ ions of m/z 363 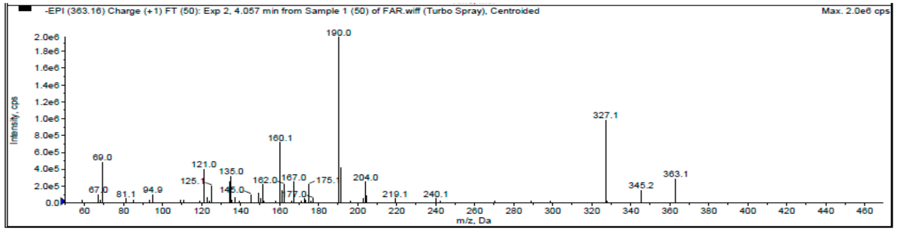	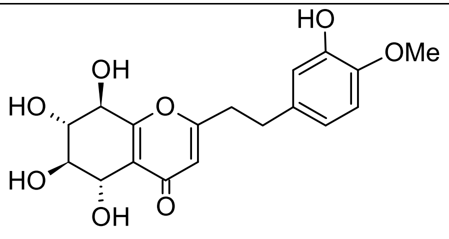 or 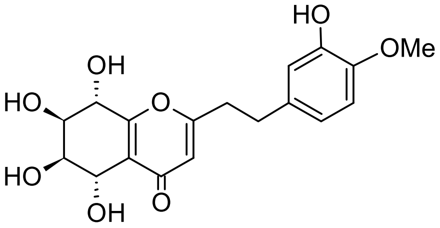
7	aquilarone B	[M-H]$^-$ ions of m/z 317 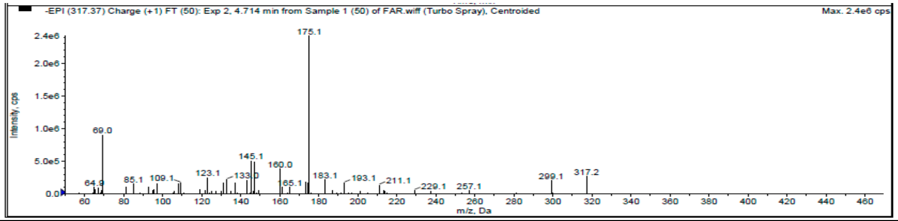	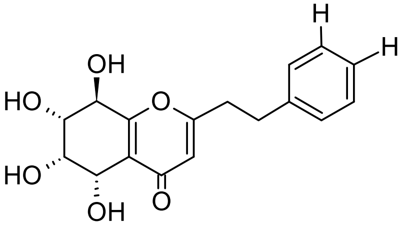

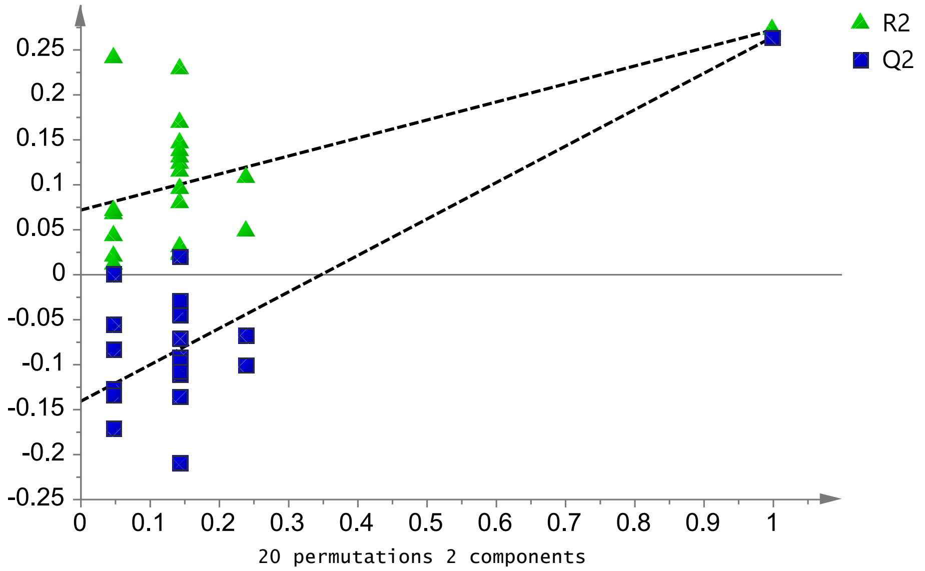

Supplementary Fig. 1 Permutation test results for the PLS-DA model developed for the *Aquilaria* gaharu samples

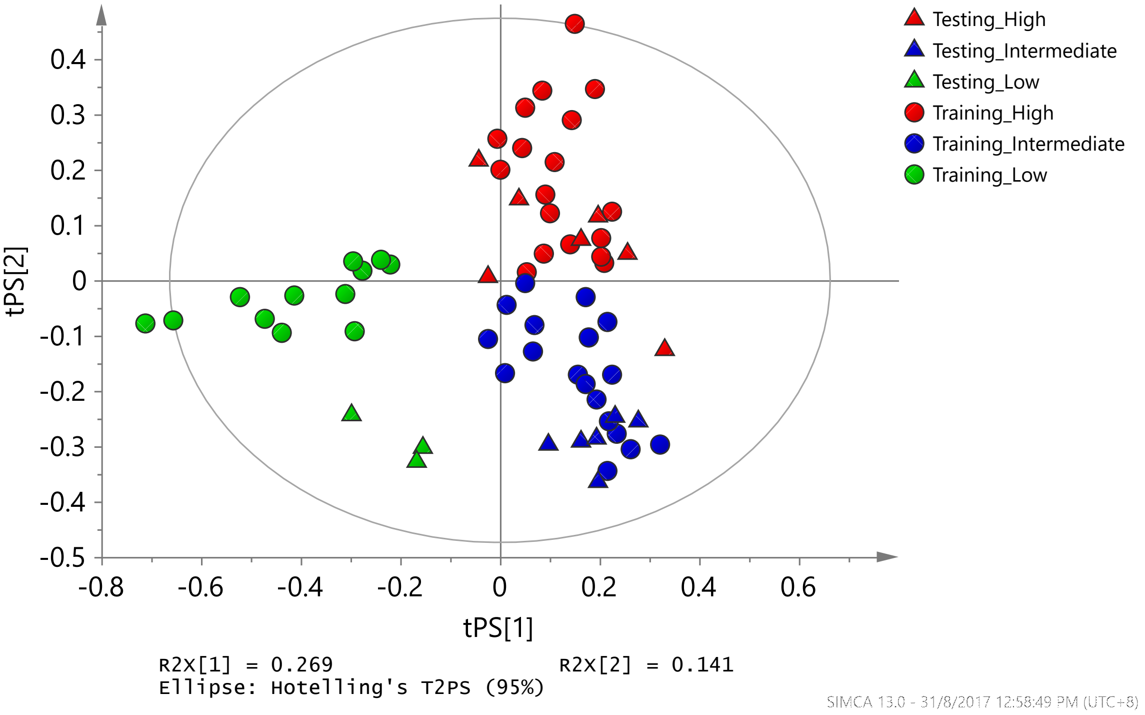

Supplementary Fig 2. PLSDA score plot of training and testing datasets of three (high, intermediate, and low) grades of gaharu samples.

(A) VIP values for PLS-DA

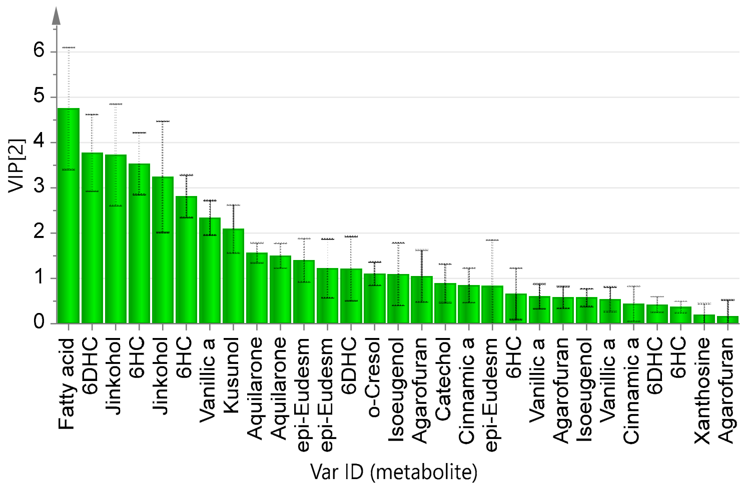

(B) VIP values for Random Forests

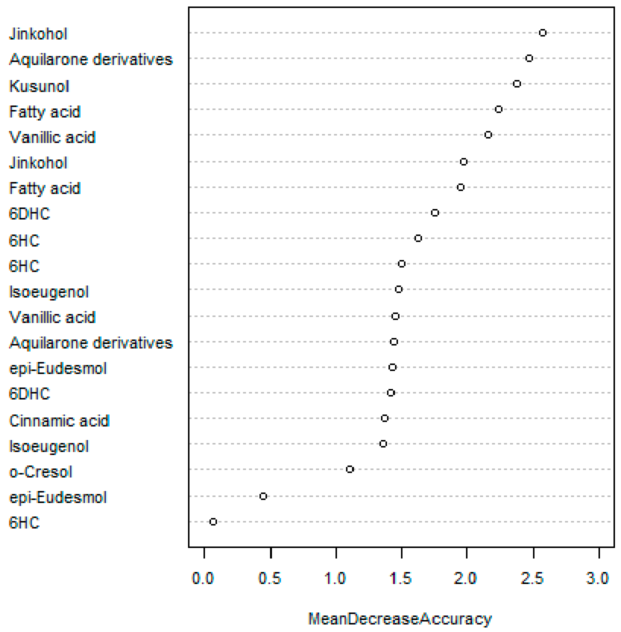

Supplementary Fig. 3 Variable importance (VIP) values for (a) PLS-DA and (b) Random Forests models

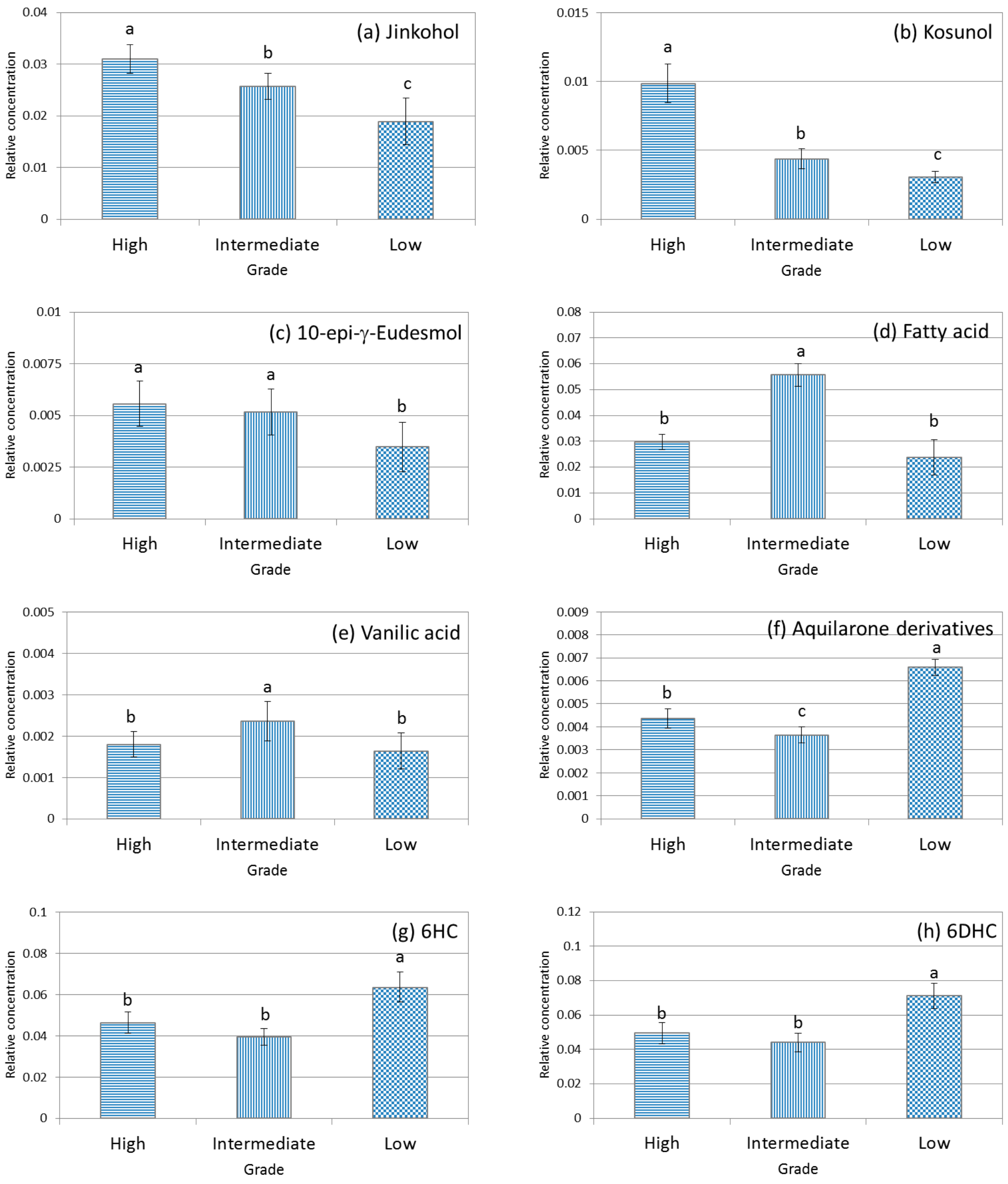

Supplementary Fig. 4 Relative quantification and statistical analysis for eight chemical markers: (a) jinkohol, (b) kosunol, (c) 10-epi-γ-eudesmol, (d) fatty acid, (e) vanilic acid, (f) aquilarone derivatives, (g) 6-hydroxy-2-(2-phenylethyl)chromone (6HC) and (h) 6-hydroxy-2-[2-(4-hydroxyphenyl)ethyl]chromone (6DHC) for high, intermediate, and low grade groups.
